# Supplementary material for: Netcombin: An algorithm for constructing optimal phylogenetic network from rooted triplets
Source: PLoS One. 2020 Sep 18;15(9):e0227842. doi: 10.1371/journal.pone.0227842 (PMC7500971; doi:10.1371/journal.pone.0227842)
Supplement: S4 File — (ZIP) [file pone.0227842.s004.zip › SequenceData-Triplet/sequenceData-10-20-30-0-Triplet/length 200, numseq 40, rate 10^-7/1 seq.pdf]

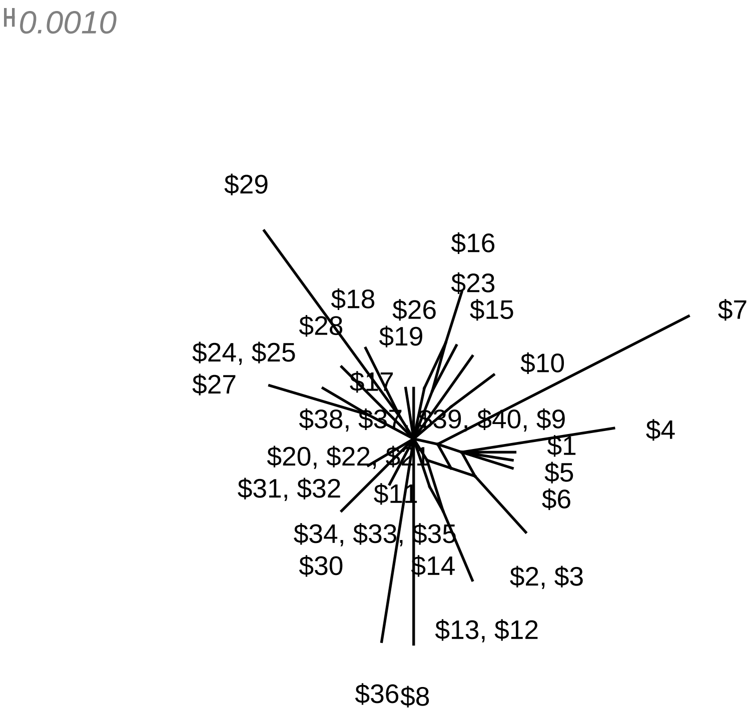
0.0010
$29
$16
$23
$18
$26
$15
$7
$28
$19
$24, $25
$10
$27
$17
$38, $37
$39, $40, $9
$4
$1
$20, $22, $21
$5
$31, $32
$11
$6
$34, $33, $35
$30
$14
$2, $3
$13, $12
$36
$8
